# Smoker perceptions of health warnings on cigarette packaging and cigarette sticks: A four-country study

**DOI:** 10.18332/tid/104753

**Published:** 2019-03-28

**Authors:** Aaron Drovandi, Peta-Ann Teague, Beverley Glass, Bunmi Malau-Aduli

**Affiliations:** 1 College of Medicine and Dentistry, James Cook University, Townsville, Australia

**Keywords:** health behavior, health promotion, public health, tobacco control

## Abstract

**INTRODUCTION:**

Innovations in tobacco control interventions are required to ensure continued reductions in global tobacco use, and to minimise attributable morbidity and mortality. We therefore aimed to investigate the perceived effectiveness of current cigarette packaging warnings and the potential effectiveness of cigarette-stick warnings across four countries.

**METHODS:**

An online survey was distributed to adult smokers in Australia, Canada, the United Kingdom, and the United States. Participants rated (using a 5-point Likert scale) and commented on the effectiveness of current cigarette packaging warnings and text warnings on eight cigarette sticks that prompted smokers to quit. Ratings were analysed using proportional odds logistic regression, and comments were analysed using content analysis.

**RESULTS:**

Participants (N=678, mean age=44.3 years) from all four countries perceived cigarette packaging warnings as being minimally effective in prompting smokers to quit, citing desensitisation and irrelevance of the warnings, with US participants particularly critical of the text-only warnings. Compared to packaging warnings, the cigarette-stick warnings describing the financial costs of smoking and the effect of smoking on others, were the highest rated in all four countries (OR=3.42, 95% CI: 2.75–4.25, p<0.001 and OR=2.85, 95% CI: 2.29–3.55, p<0.001, respectively) and cited as strong messages to reduce smoking. Half of the participants either ‘agreed’ or ‘strongly agreed’ to the use of cigarette-stick warnings.

**CONCLUSIONS:**

The findings of this study suggest that cigarette packaging warnings may experience a loss of effectiveness over time, eventually resulting in minimal impact on smoker behaviour. Health and non-health focused warnings and messages on individual cigarette sticks represent a novel and potentially effective method for reducing tobacco use. This would complement tobacco control interventions currently employed, resulting in public health benefits.

## INTRODUCTION

Tobacco use remains the largest cause of preventable morbidity and mortality^1^, despite the majority of smokers regretting smoking and wanting to quit^2,3^. Quit intentions are influenced by multiple factors, particularly the financial cost of smoking^1,4^ and awareness of the negative health consequences of tobacco use^4,5^. Messages portraying these consequences are often prominently conveyed in developed countries through a combination of mass media campaigns and cigarette packaging warnings^6,7^. The World Health Organization (WHO) Framework Convention on Tobacco Control (FCTC) details minimum recommendations for these public health interventions. FCTC Article 11 describes recommended packaging and labelling of tobacco products, including the use of text and pictorial warnings, plain packaging, and the removal of misleading branding elements^8^.

Within the Health Belief Model (HBM), health-related behaviours can be explained and predicted through a person’s values and expectations from performing these behaviours^9^. Key elements within this model (as applicable to smoking) include a person’s perceived susceptibility to and severity of smoking-related consequences, perceived benefits of and barriers to quitting, and the cues to action in changing smoking behaviours and self-efficacy in doing so^10^. A significant volume of research has found that tobacco packaging interventions have been effective in addressing gaps in knowledge on the dangers of smoking and misconceptions of cigarette safety, and enhancing the perceived susceptibility and severity of smoking-related consequences^7,11-16^. This includes large international studies such as the International Tobacco Control (ITC) Policy Evaluation Project that evaluated the effectiveness of various tobacco control policies in 29 countries, including the effectiveness of health warnings in Australia, Canada, the USA, and UK^3,12,16^. There are, however, few messages on cigarette packaging that either reinforce the benefits of quitting (especially non-health related benefits) or improve smoker self-efficacy in quitting smoking^15^. Whilst these interventions have overall led to significant decreases in tobacco use, they may be subject to a ‘wearing-out’ effect due to repeated exposures, with regular smokers viewing these health warnings thousands of times per year^16-18^. This suggests the need for frequent changes of tobacco packaging interventions to ensure continued impact on smoking behaviour.

Recent research has identified the cigarette stick as a potentially effective medium for conveying the risks of smoking, and may complement warnings present on cigarette packaging^19-24^. As the primary packaging of tobacco, cigarette sticks represent a logical and visible medium for health warnings^25^. Initial cigarette-stick warnings, evaluated amongst smokers and non-smokers, were limited and included ‘Smoking Kills’, the ‘Minutes of Life Lost’ per cigarette, and a list of toxic cigarette constituents^19-25^. These preliminary studies received positive responses from participants but further research is needed to better evaluate the potential effectiveness of this form of public health intervention^19-26^. Subsequent research investigating the potential effectiveness of a wider range of health and non-health warnings also reported positive findings, with high perceived effectiveness ratings among several warnings and agreeability towards cigarette-stick warnings^27-29^. However, these studies largely involved non-smoking Australian participants.

Therefore, this study aimed to expand upon previous research on cigarette-stick warnings, using a smoker cohort. We first aimed to evaluate the perceptions of an international cohort of smokers on the effectiveness of cigarette packaging warnings, to identify their strengths and weaknesses as a tobacco control intervention. We then aimed to evaluate the perceptions of these smokers towards eight health warnings and messages on individual cigarette sticks, and identify those considered most effective in influencing smoker behaviours. Building on the findings of earlier studies^27-29^, we intended to assess how perceptions towards cigarette packaging and cigarette-stick warnings could lead to the development of effective cigarette-stick interventions that supplement packaging warnings, and overcome the weaknesses of packaging warnings as identified by participants. Finally, we also aimed to gauge participants’ support towards the inclusion of health warnings on individual cigarettes as a public health intervention to reduce tobacco use.

## METHODS

### Study design and participant recruitment

This study used a cross-sectional design with an online survey, distributed to adult smokers in Australia, Canada, the United States, and the United Kingdom, in June 2018 using the ‘targeted audience’ function in SurveyMonkey. This function allows surveys to be distributed to specific participants, which for this study were smokers over the age of 18 years, who use cigarettes in these four countries. We used the targeting function to recruit 150 smokers from each country, who received no remuneration for participation. This desired minimum participation number was chosen based on the observed significance of results from previous research on this topic^27-29^ as well as on financial limitations. Previous research utilising similar interventional materials of the current study primarily recruited Australian non-smokers^27-29^. Therefore, we aimed in the present study to assess if these findings were relevant to smokers, and had international applications as a tobacco control method. Three additional countries were chosen for participant recruitment in order to include those countries that examined the effectiveness of health warnings in the ITC Project, which have similar tobacco control interventions and have English as their primary language in order to prevent translational issues for the intervention materials. This research was approved by the James Cook University Human Research Ethics Committee (approval number H6929).

### Procedure and data items collected

**Figure 1:**
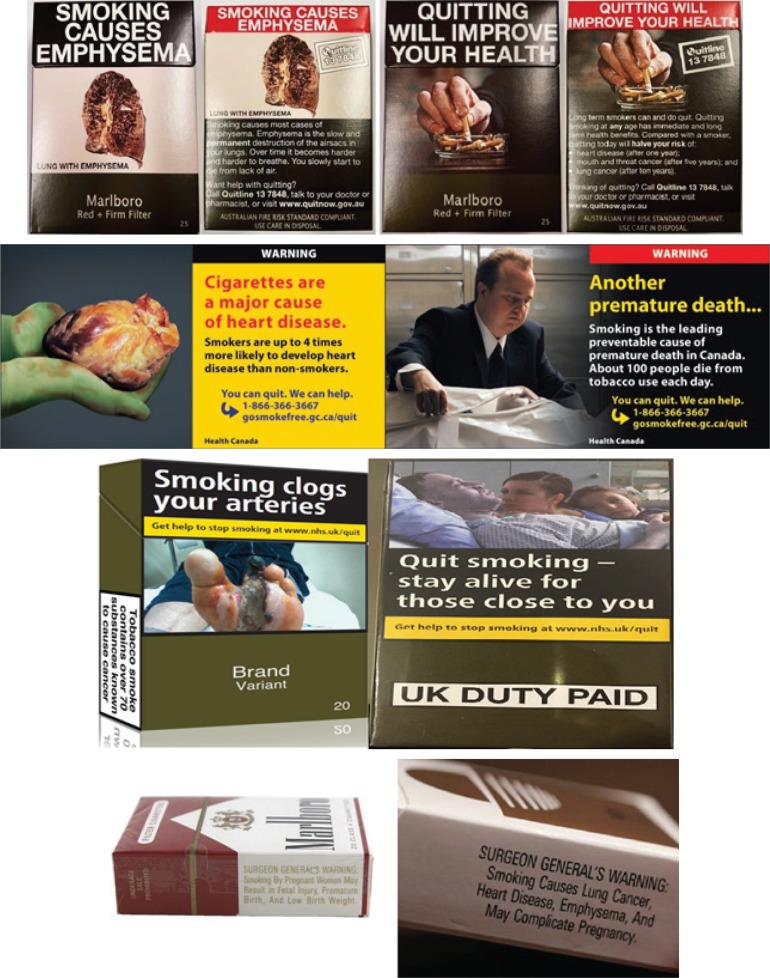
Cigarette packaging warnings displayed to participants from each country: Australia (top), Canada (second), United Kingdom (third), United States (bottom)

The first part of the survey requested sociodemographic information that included: participant country of origin, gender, age, ethnicity, level of education, cigarettes smoked per day (CPD), intentions to quit smoking, and baseline perceptions of the level of harm caused by smoking. The survey had four country-specific versions to account for major ethnic backgrounds in each country, and for some of the interventional materials. Participants were then shown two country-specific cigarette packaging warning examples, representative of the main themes of tobacco control messages used in their country (Figure 1), and asked to rate on a 5-point Likert primarily recruited Australian non-smokers^27-29^.

Therefore, we aimed in the present study to assess if these findings were relevant to smokers, and had international applications as a tobacco control method. Three additional countries were chosen for participant recruitment in order to include those countries that examined the effectiveness of health warnings in the ITC Project, which have similar tobacco control interventions and have English as their primary language in order to prevent translational issues for the intervention materials. This research was approved by the James Cook University Human Research Ethics Committee (approval number H6929).

### Procedure and data items collected

The first part of the survey requested sociodemographic information that included: participant country of origin, gender, age, ethnicity, level of education, cigarettes smoked per day (CPD), intentions to quit smoking, and baseline perceptions of the level of harm caused by smoking. The survey had four country-specific versions to account for major ethnic backgrounds in each country, and for some of the interventional materials. Participants were then shown two country-specific cigarette packaging warning examples, representative of the main themes of tobacco control messages used in their country (Figure 1), and asked to rate on a 5-point Likert scale (from ‘Not at all effective’ to ‘Very effective’) the effectiveness of these warnings in prompting them to quit. They were also prompted to detail in open-text comment boxes on specific strengths or shortcomings of cigarette packaging warnings used in their respective country. Ratings and comments of current packaging warnings were gathered to act as a baseline comparison against cigarette-stick warnings (described below) and to better understand how these two forms of public health intervention could work together to reduce tobacco use.

**Figure 2:**
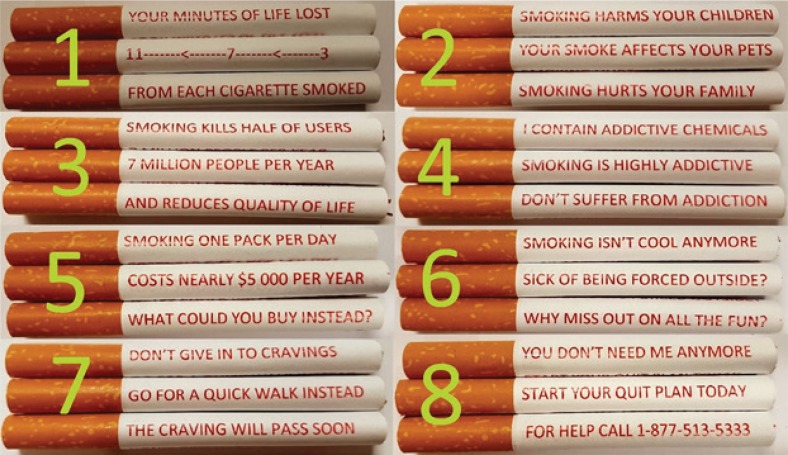
The eight cigarette-stick warnings and messages displayed to participants (in random order). Note: Cigarettes 5 and 8 were different in each of the four versions of the survey to account for country-specific differences in the financial cost of smoking (Australia: $11000, Canada: $5000, UK: £4000, USA: $2500 ) and phone numbers for help lines

Photos of eight cigarettes with messages printed in red along their shafts were then displayed. Each cigarette had three lines of text (with all sides shown per cigarette) that could be read as it was rotated, thus depicting a full message or warning relating to tobacco use (Figure 2). Participants were informed that cigarette-stick warnings could be applied using non-toxic vegetable inks. The eight cigarette-stick warnings were each presented in random order and participants then rated on a 5-point Likert scale (from ‘Not at all effective’ to ‘Very effective’) their perceived effectiveness in prompting them to quit. They also had the option of describing the reasons behind each rating in open-text comment boxes. The cigarette-stick warnings were designed based on the elements of the Health Belief Model, on previous research conducted by the authors of the present study, and on earlier studies in the UK and New Zealand on cigarette-stick warnings^19-29^. Participants were then asked to rank each of the eight cigarette-stick warnings from most to least effective. Lastly, participants rated on a 5-point Likert scale (from ‘Strongly disagree’ to ‘Strongly agree’) their support for or against the implementation of health warnings on individual cigarette sticks in their country.

### Analysis

Descriptive analysis was used to determine the sociodemographic characteristics of the study population. Non-parametric tests (Kruskal-Wallis and Mann-Whitney U) in SPSS v25 (IBM Corp. Armonk, NY, USA) were used to investigate relationships between sociodemographic variables and participant perceptions of the health warnings, with p-values set at 0.05. Friedman Test was used to measure changes in participants’ perceptions across the 9 items (current warnings and the 8 interventional cigarette warnings). Post-hoc tests and Bonferroni adjustments were used to determine statistically significant differences between the categories. Proportional odds logistic regression was used to account for the ordered categorical responses in the survey and was performed using R v33.2.4 (R Core Team, Vienna, Austria) ordinal statistical package. This allowed us to evaluate between and within-intervention effectiveness (in comparison to current packaging warnings), using the Likert-scale ratings for warning effectiveness as the dependent variable. Responses from open-text comments were analysed independently by two authors (AD and BMA) using conceptual content analysis to confirm emerging themes. This involved the identification, coding, and quantification of key concepts raised by participants relative to individual Likert-scale questions. To establish trustworthiness of the data, findings were compared and conflicting interpretations were resolved through dialogue. Illustrative quotes are reported verbatim to support the discussion.

## RESULTS

### Sociodemographic profile

Of the 717 participants who accessed the survey, 687 (96%) were eligible for inclusion and their characteristics are shown in Table 1. There were slightly more females than males (53.4% vs 46.6%), with a relatively even spread across age groups (33.9% for 18–35 years, 40.6% for 36–55 years, and 25.5% for 56 years and older). Most participants had completed high school (98.9%), were of Caucasian descent (82.2%), and smoked between one and twenty CPD (70.3%), though only half (50.4%) had plans to quit smoking. The majority (80.2%) also recognised that smoking was ‘quite’ or ‘very’ harmful to a person’s health (4 and 5, on the 5-point Likert scale, respectively).

**Table 1:** Participant sociodemographics for each country (total N=687 )

	*Australia*	*Canada*	*United Kingdom*	*United States*	*Total (%)*
Number of participants, n	190	165	155	177	687
**Gender**					
Male	77	89	67	87	320 (46.6)
Female	113	76	88	90	367 (53.4)
**Age** (years)					
18–35	65	81	60	27	233 (33.9)
36–55	99	58	63	59	279 (40.6)
≥56	26	26	32	91	175 (25.5)
Mean	41.4	39.1	42.5	53.8	44.3
Range	19–73	18–78	19–74	19–84	18–84
Standard Deviation	12.8	14.0	13.4	14.8	14.9
**Ethnicity**					
Caucasian	159	120	139	147	565 (82.2)
Indigenous*	11	11	9	8	39 (5.7)
Hispanic	0	0	0	11	11 (1.6)
Asian	10	27	3	7	47 (6.8)
African	1	2	1	0	4 (0.6)
Middle-Eastern	6	2	2	3	13 (1.9)
No response	3	3	1	1	8 (1.2)
**Education**					
No schooling	0	1	0	2	3 (0.4)
Primary school	2	2	0	1	5 (0.7)
High school	54	55	57	44	210 (30.6)
Trade/Vocational	66	36	34	47	183 (26.6)
Undergraduate	51	47	37	52	187 (27.2)
Postgraduate	17	24	27	31	99 (14.4)
**Cigarettes per day**					
Less than daily	24	19	14	22	79 (11.5)
1–10	57	60	58	56	231 (33.6)
11–20	63	57	57	75	252 (36.7)
21–30	35	22	20	16	93 (13.5)
≥31	11	7	6	8	32 (4.7)
**Intentions to quit**					
No intentions	24	21	20	30	95 (13.8)
Intends to (no plan)	67	60	55	67	249 (36.2)
Within 12 months	80	65	59	30	237 (34.5)
Within 3 months	19	19	21	50	109 (15.9)
**Perceptions of harm from smoking**					
Not at all harmful	1	2	1	0	4 (0.6)
Minimally harmful	4	8	4	3	19 (2.8)
Moderately harmful	34	26	19	34	113 (16.4)
Quite harmful	51	57	45	66	219 (31.9)
Very harmful	100	72	86	74	332 (48.3)

* Australia: Aboriginal or Torres Strait Islander; Canada: Native Canadian or African American; United Kingdom: Black British or Afro-Caribbean; United States: African American. Exact Indigenous numbers per country are available in Supplementary Appendix 2.

### Perceived effectiveness of current cigarette packaging warnings

Cronbach alpha for the Likert-scale questions was 0.94, indicating very high internal consistency. There were significant differences between most countries for participant ratings of current packaging warning effectiveness. US participants had significantly lower ratings for their packaging warnings compared to those of other countries (p<0.05), with nearly three-quarters (72.3%) of participants considering them ‘not at all’ or ‘minimally’ effective (1 and 2, on the Likert scale, respectively) in prompting current smokers to quit (mean rating 2.07). This is in comparison to half (51%) of Australians (mean rating 2.54), and one-third (36.1% and 35.1%) of UK (mean rating 2.98) and Canadian (mean rating 2.87) participants, respectively (χ^2^=83.177, p<0.001).

Other factors associated with the ratings of current packaging warnings included participant age, and the number of CPD smoked. The youngest age group were more likely to consider them as ‘quite’ or ‘very’ effective (4 and 5, on the 5-point Likert scale, respectively) compared to the oldest participants (27.9% vs 14.3%, χ^2^=18.904, p= 0.015). Similarly, lighter smokers were more likely to consider them as ‘quite’ or ‘very’ effective (38% for occasional smokers, and 26.8% for those smoking 1 to 10 CPD), compared to heavier smokers (18.3% for those smoking 21–29 CPD, and 15.7% for those smoking 30 or more CPD) (χ^2^=38.887, p<0.001).

The open-text comments reflected the Likert-scale ratings, with nearly two-thirds (63.4%) of participants describing their opinions of current packaging warnings. Whilst some participants from each country (from 10.2% for US up to 23.7% for Australia) described the warnings as retaining some of their efficacy (particularly on youth), the majority of comments (from 48.0% of participants) were negative. The most common reasons for negative comments on the effectiveness of current packaging warnings were a perceived loss of efficacy, warning irrelevance to smokers (especially younger participants), or desensitisation towards the warnings. These comments were made by many participants, ranging from 33.3% of Canadians up to 41.6% of Australians.

*‘Because they’re everywhere, people become desensitised to them. I know they don’t bother me anymore.’* (Female, 30, Australia).

*‘Worst case scenario portrayed on packs, minimal effect on quitting’* (Male, 63, UK).

*‘If someone is willing to smoke, they will smoke no matter what the message or image on the packet is.’* (Male, 36, Canada).

*‘I think that when originally implemented, the impact was very much higher than today. I also suspect that even today the warnings will have some effect on youth.’* (Male, 70, US).

### Perceived effectiveness of health warnings on cigarette sticks

Cigarette stick warnings were rated in the same country-specific order as for the cigarette packaging warnings, with UK smokers rating each cigarette stick the highest, followed by those from Canada then Australia, and US giving the lowest ratings (Supplementary Appendix 1 and Figure 2). Compared to the overall mean rank for packaging warnings (2.60 out of 5), cigarette-stick ratings 1 to 8 were 2.91, 3.06, 2.93, 2.61, 3.11, 2.49, 2.53, and 2.72, respectively. Table 2 shows the results of the proportional odds logistic regression analysis, including reference levels and points of significance. The cigarette warning describing the financial costs associated with smoking (cigarette 5, Figure 2) was consistently rated the most effective in all four countries (OR=3.42, 95% CI: 2.75–4.25, p<0.001) compared to current packaging warnings followed by the cigarette warnings describing the effect of smoking on others (cigarette 2, Figure 2) (OR=2.85, 95% CI: 2.29–3.55, p<0.001). The lowest rated cigarette warning overall (cigarette 6, Figure 2) describing social issues associated with smoking, was rated lowest in all countries except for the UK (where it was the second lowest) (OR=0.70, 95% CI: 0.57–0.88, p=0.002). Other factors associated with cigarette-stick ratings included: age, CPD, and quit intentions. The oldest age group were less likely to rate cigarettes 1, 2, 5 and 7 (Figure 2), as ‘quite’ or ‘very’ effective compared to the youngest age group (all p<0.01). Heavier smokers similarly were significantly less likely to rate cigarettes 1–6 (Figure 2) as effective compared to occasional smokers (all p<0.01), as were those with no quitting intentions compared to those who had plans to quit within the next 12 months, for all 8 cigarette warnings (all p<0.01).

**Table 2:** Proportional odds logistic regression model, with p-values in bold showing points of significance within the data

*CHARACTERISTICS*	*VARIABLE*					*p*
	*Est.*	*SE*	*Z*	*OR*	*95% CI*	
Gender (Female=0, Male=1)	0.487	0.219	2.219	1.63	1.06–2.50	0.026*
**Age group** (years)^a^						
36–55	-0.274	0.255	-1.075	0.76	0.46–1.25	0.283
≥56	-0.428	0.310	-1.380	0.65	0.36–1.20	0.168
**Ethnicity^b^**						
Indigenous	1.307	0.465	2.812	3.70	1.49–9.19	0.005**
Asian	0.426	0.444	0.961	1.53	0.64–3.66	0.337
Other	0.249	0.484	0.515	1.28	0.50–3.31	0.607
**Education^c^**						
Trade/Tech/Voca	-0.075	0.281	-0.268	0.93	0.53–1.61	0.789
Undergraduate	-0.256	0.282	-0.909	0.77	0.45–1.35	0.363
Postgraduate	-0.364	0.345	-1.054	0.69	0.35–1.37	0.292
**Country**^d^						
Canada	0.469	0.301	1.560	1.60	0.89–2.88	0.119
UK	0.853	0.304	2.811	2.35	1.29–4.26	0.005**
USA	-0.388	0.321	-1.208	0.68	0.36–1.27	0.227
**Quit intentions^e^**						
No plans to quit	-0.082	0.346	-0.237	0.92	0.47–1.82	0.813
<12 months	0.865	0.358	2.417	2.38	1.18–4.79	0.016*
<3 months	0.645	0.415	1.556	1.91	0.85–4.30	0.120
**Cigarettes per day^f^**						
1–10	-0.549	0.365	-1.506	0.58	0.28–1.18	0.132
11–20	-0.482	0.363	-1.327	0.62	0.30–1.26	0.185
≥21	-1.636	0.412	-3.975	0.19	0.09–0.44	<0.001***
**Perceptions of harm caused by smoking^g^**						
Quite harmful	0.769	0.313	2.458	2.16	1.17–3.98	0.014*
Very harmful	1.270	0.303	4.195	3.56	1.97–6.45	<0.001***
**Cigarette-stick effectiveness^h^**						
Financial cost of smoking	1.230	0.111	11.091	3.42	2.75–4.25	<0.001***
Effect of smoking on others	1.048	0.111	9.484	2.85	2.29–3.55	<0.001***
Risk of mortality from smoking	0.764	0.110	6.978	2.15	1.73–2.66	<0.001***
Minutes of life lost	0.725	0.110	6.572	2.06	1.66–2.56	<0.001***
Planning to quit	0.222	0.110	2.016	1.25	1.01–1.55	0.044*
Risk of addiction from smoking	-0.055	0.110	-0.505	0.95	0.76–1.17	0.614
Dealing with cravings	-0.227	0.111	-2.043	0.80	0.64–0.99	0.041*
Social issues with smoking	-0.351	0.111	-3.166	0.70	0.57–0.88	0.002**

a Reference level was the 18–35 years age group.

b Reference level was Caucasian heritage.

c Reference level was High School education.

d Reference level was Australia.

e Reference level was no interest or intentions to quit.

f Reference level was occasional smoking.

g Reference level was ‘Some Harm’ (3 on Likert Scale).

h Reference level was current packaging warnings.

*** p<0.001

** p<0.01

* p<0.05; p-values in bold significant.

There were fewer open-text comments provided for cigarette-stick warnings (between 12% and 15% of participants per cigarette), though these comments provided insight as to why certain warnings and messages were perceived as more effective than others. Comments for the cigarette describing the financial costs of smoking were evenly split between those that were supportive/positive, and those that were dismissive/negative. Positive comments described the importance of money as a motivator for quit attempts, with the large annual cost associated with smoking as being a powerful message. *‘When you bring the financial aspect into it, it really opens people’s eyes and they might cut down or even quit.’* (Male, 30, Canada).

*‘This is the most effective argument of all. People play fast and loose with health issues, but a reminder about the drain on the wallet will probably be a lot more effective with many people in our current times.’* (Female, 47, US).

Negative comments related to warning irrelevance (e.g. to smokers who smoked less than one pack per day) or already being aware of the financial cost of smoking.

*‘There are a lot of smokers who do not smoke that much, that this wouldn’t persuade.’ (Female, 22, Canada). ‘People are aware of the cost of cigarettes when they go buy them, and this doesn’t change their view.’* (Female, 22, UK).

Comments for the cigarette warning describing the effect of smoking on others were slightly more positive (58% vs 42%), with participants usually acknowledging the importance of not harming others as a result of their habit, though many cited the irrelevance of the warnings to their personal situation, or that they knew about the effects of smoking on others and had already taken steps to prevent this issue.

*‘If you care about your family and pets, especially young children, how can you ignore this one?’* (Female, 60, US).

*‘I smoke outside to avoid this, so it doesn’t affect me.’* (Female, 22, UK).

*‘Family is probably the biggest concern for me, and that they may have to deal with the consequences of my habit.’* (Male, 26, Australia).

*‘I have no children or pets, and I only smoke around family that smoke.’* (Male, 48, Canada).

### Support for health warnings on cigarette sticks

The same country-specific order as seen previously was seen for including health warnings on individual cigarette sticks, with UK smokers being the most supportive, followed by Canada, Australia, and the United States. Participant acceptance of the implementation of cigarette-stick warnings was high for each country, with about half (50.7%) of all participants either ‘agreeing’ or ‘strongly agreeing’. Country-specific averages were 3.31, 3.43, 3.75, and 3.91 out of 5 for Australia, Canada, the UK, and United States, respectively, with a total average of 3.42 out of 5. Only 12% of participants left open-text comments (likely due to being the end of the survey), though this included strong and emotive responses equally for and against warnings on cigarette sticks.

*‘I think this would have teenagers thinking twice, I know it would have impacted me greatly as a teen. Even now as an adult we all need constant reminders in our lives to do better, and I think these statements do it way better than the old ads.’* (Female, 35, US).

*‘Printed comments on the actual cigarette seems like a joke. If they have a cigarette in their hand they are* going to smoke it no matter what is printed on it, just like on the box.’ (Female, 42, US).

*‘Everyone is used to seeing the warnings on the packages and most often those packages are thrown away. It could be different if the warnings were on individual cigarettes.’* (Female, 24, Canada).

*‘Smokers are immune to pictures and words. I couldn’t even tell you what is on the packet I’m smoking now.’* (Female, 54, Australia).

Other factors associated with support for cigarette-stick warnings included intentions to quit smoking, and baseline perceptions of the harms of smoking. Those who intended to quit smoking, and those acknowledging the dangers of smoking were more likely to ‘agree’ or ‘strongly agree’ to the inclusion of health warnings on cigarette sticks compared to those with no intentions to quit, and those who only considered smoking ‘somewhat harmful’ (p<0.001).

## DISCUSSION

In this study, health warnings on cigarette packaging currently implemented across Australia, Canada, the United Kingdom, and the United States were generally perceived as minimally effective in prompting current smokers to quit, with irrelevance and desensitisation to the warnings being commonly cited. In comparison, four of the eight cigarette-stick warnings were rated as more effective than current packaging warnings in all countries (cigarettes 1, 2, 3 and 5, Figure 2), with US participants rating all eight cigarette warnings higher than their current packaging warnings. Within the HBM, these four cigarette-stick warnings all aimed to increase readers’ perceived susceptibility and severity of smoking. There was also significant support for the inclusion of health warnings on individual cigarette sticks, with half of participants ‘agreeing’ or ‘strongly agreeing’ with the premise. Based on the findings of this study, we believe that cigarette-stick warnings might serve as an effective supplementary public health intervention, particularly if the messages delivered relate to those present on cigarette packaging. The novelty and visibility of cigarette-stick warnings are key aspects expected to lend to their ability to communicate the consequences of smoking.

Of the four participating countries in this study, the US is the only one without pictorial warnings on its packaging, including only small text warnings^15^, likely responsible for the lower ratings of packaging warning effectiveness compared to the other countries. These findings reinforce the need for more effective tobacco packaging interventions in the US, such as those initially planned for release in 2012 but prevented through an injunction initiated by several tobacco companies^30^. Also, despite having pictorial warnings present in Australia, ratings of packaging warnings were lower than those of Canada and the UK. This could be potentially due to differences in the variety of warning themes and specific pictures used in Australia compared to Canada, and the recent implementation of plain packaging in the UK, which increases the visibility and recall of warnings^15^. Two common themes expressed by participants in all four countries were perceived irrelevance and desensitisation to the warnings, demonstrating the need for warnings that are both novel and more applicable to the wider population. Desensitisation is a well-recognised issue, which several countries have attempted to minimise, through techniques such as rotating sets of warnings (Australia), supplementary packaging inserts (Canada), and using plain packaging (in progress in various countries)^31,32^.

To combat these issues, we found that less emphasis on the ‘worst-case’ or ‘end-game’ diseases that might result from smoking (such as those currently dominating cigarette packaging warnings) and a greater emphasis on negative outcomes (and not those restricted to personal health) that affect a wider proportion of smokers earlier in their smoking career may have greater impact. For example, we identified the financial consequences of smoking as being consistently perceived as the most effective message by participants in prompting current smokers to quit, a message that is not currently portrayed on cigarette packaging in any of the four participating countries, despite research identifying it as a key motivator for quit attempts^1,4,33-36^. Within the HBM, and as identified through the open-text comments, perceived susceptibility and severity of the financial strains of smoking appear to be much more generalizable and relatable than health-related consequences of smoking. Similar to the shortcomings of current packaging warnings, perceived irrelevance may have limited the ratings of this financial message, as nearly half of participants smoked half a pack per day or less, reducing the impact of the annual cost estimate of smoking one pack per day. Increased message relatability (and effectiveness) could be achieved through depicting the fortnightly or monthly costs of light or moderate smoking, which are shorter-term and may be more relatable in terms of general living costs. Implementing such a message would be particularly beneficial if used in conjunction with tax increases on tobacco products, such as those being annually applied within Australia^37^.

Unlike the financial costs of smoking, the second highest rated warning in this study describing the effects on others has been implemented on cigarette packaging (except US), indicating the need for this message theme to continue as a tobacco control intervention. Many participants considered this warning irrelevant to them, particularly if they were already taking steps to minimise the exposure of those around them to their smoking, though previous research has indicated that not all smokers acknowledge that smoking can cause significant harm to nearby non-smokers^11,13,38^. Improving public awareness of the effects of both secondhand and thirdhand smoke may lead to improved efficacy for this warning theme^38,39^. Previous research has also identified a gap in knowledge on the specific health consequences of tobacco use^11,13^. Whilst not explicitly examined in this study, some lesser-acknowledged consequences of tobacco use, such as male impotence, earlier onset of menopause, osteoporosis and several dental diseases may benefit from greater exposure on both cigarette packaging and cigarette sticks, potentially made more effective through embarrassment or guilt when visible to onlookers^11,13,40,41^. A similar effect occurs with dissuasively coloured cigarettes with darker coloured cigarette paper, opposing the desired persona of smokers, increasing their perceptions of the cigarette in causing harm, and stimulating quit attempts^21,26^. An investigation into combining dissuasively coloured cigarettes and cigarette-stick warnings would be an important next step in evaluating the full potential of the cigarette stick as a tool for controlling tobacco use.

Increasing smokers’ perceived susceptibility to both health and non-health consequences of smoking, through a combination of cigarette packaging and cigarette-stick warnings and messages, is likely to prompt quit attempts amongst smokers. An additional advantage of cigarette-stick warnings is their visibility during smoking, and inability to be easily concealed or avoided entirely, as can occur for packaging warnings, particularly amongst adolescents^42,43^. The severity of the consequences portrayed should also be perceived as applicable to the majority of smokers, which was identified as a limitation of current packaging warnings and of some cigarette-stick warnings in this study. Apart from these two components, which are commonly addressed through current packaging warnings, the Health Belief Model also indicates the significance of a smokers’ cue to action and self-efficacy in quitting9. Cigarettes 7 and 8 (Figure 2), which give advice on how to quit and deal with cravings were rated similarly to packaging warnings, though previous research has indicated that some adult smokers prefer this approach and encourage the availability of supportive messages^44,45^.

Amongst this cohort, younger and lighter smokers demonstrated higher perceived effectiveness ratings towards both cigarette packaging and cigarette-stick warnings, likely due to their less extensive dependence on tobacco products and exposure to packaging warnings, alongside recent trends of improved public health initiatives as per the WHO FCTC guidelines^8^. Additionally, older participants believed that packaging warnings, whilst ineffective in themselves, retained some efficacy on youth, and supported both packaging and stick warnings aimed at encouraging young smokers to quit, and non-smokers from experimenting with tobacco. Adolescents and young adults form a key target group, both for advertising by tobacco manufacturers^46^ and public health interventions^47^, as they represent the next generation of potential smokers. It is therefore essential that new public health interventions, such as cigarette-stick warnings, include messages that appeal to this vulnerable age group. However, warning irrelevance of current packaging interventions amongst this age group has been found to be an issue in similar studies^28,29^, prompting the need for regularly updated warnings.

As yet, no country has implemented cigarette-stick warnings, though the Canadian government’s public health department recently issued a call for consultation on new health labelling for tobacco products that included cigarette-stick warnings^48^. To move cigarette-stick warnings from theory into practice, further research on a larger international population of smokers and non-smokers, using tailored and generalizable health warnings and messages, is needed to better determine the potential efficacy of this novel form of intervention. Regular updates and message rotation would also require investigation, to ensure that cigarette-stick warnings do not suffer from the same loss of impact over time as packaging warnings^12,49^. Longitudinal studies are also needed to assess the effects of repeated exposures to the intervention materials and the resulting change in perceptions and behaviours over a longer period. Identifying specific reactions to individual warnings, such as their ability to attract attention, comprehension, credibility, emotional appeal, and personal applicability, would provide more detail as to why certain warnings are perceived as effective and how ineffective warnings may be improved.

### Limitations

Limitations to consider when interpreting our results include that the participants were solely from developed countries whose tobacco packaging warnings and policies differ from those of developing countries. This includes differences in smoking prevalence, social acceptability, and the rates of use of non-cigarette tobacco products. Participants’ history of smoking (number of years) was not gathered in this study, and might have had a significant effect on their perceptions. Comparing cigarette-stick and cigarette packaging warnings is also made difficult when taking into account the medium of warning delivery, with the novelty of cigarette-stick warnings likely influencing to some extent the Likert-scale ratings of warning effectiveness. The presentation of different packaging warnings per country prior to the stick warnings may have also conditioned participants and influenced their ratings of the cigarette-stick warnings. We also did not compare the sociodemographics of the samples against the norm for each country, with sample bias potentially affecting the generalisability of the findings to each country and also to countries not involved in this study. The brief exposure to each warning also did not replicate real-world situations, or examine the diminishing effectiveness of warnings over repeated exposures. The use of online photographs compared to tactile materials may have also affected participant responses.

## CONCLUSIONS

This study identified current health warnings on tobacco packaging in four countries as having lost their impact as deterrents to smoking, highlighting the need for an update in current tobacco packaging interventions. We also found that health warnings and messages on cigarette sticks were generally well-received, and perceived as an effective additional source of information for smokers, particularly those that relate to the financial burdens of tobacco use and the effect that smoking has on others apart from the active smoker. Providing novel and effective messages for smokers to prompt quit attempts could result in significant public health benefits through the reduction of tobacco-attributable morbidity and mortality.
