# Supplementary material for: Smoker perceptions of health warnings on cigarette packaging and cigarette sticks: A four-country study
Source: Tob Induc Dis. 2019 Mar 28;17:23. doi: 10.18332/tid/104753 (PMC6751965; doi:10.18332/tid/104753)
Supplement: Supplementary file 1 [file TID-17-23-s1.pdf]

Supplementary Appendix 1. Country-Specific Likert-Scale Ratings of Cigarette-Packaging and Cigarette-Stick Health Warnings.

**Country**	**Current**	**Cig 1**	**Cig 2**	**Cig 3**	**Cig 4**	**Cig 5**	**Cig 6**	**Cig 7**	**Cig 8**	**Cig AVG**	**OpinHW**
**AUS**	2.54	2.79	3.04	2.85	2.46	**3.11**	**2.34**	2.51	2.63	2.72	3.31
**CAN**	2.87	3.08	3.18	3.02	2.75	**3.27**	**2.56**	2.63	2.85	2.91	3.43
**UK**	2.98	3.17	3.28	3.25	2.89	**3.30**	2.82	**2.74**	2.92	3.05	3.75
**USA**	2.07	2.65	2.77	2.66	2.38	**2.78**	**2.29**	2.29	2.53	2.55	3.19
**AVG***	2.60	2.91	3.06	2.93	2.61	**3.11**	**2.49**	2.53	2.72	2.79	3.42

*AVGs may be slightly different due to rounding done in excel rather than with 2 decimal points.

Bold and green = Highest scoring cigarette per country (financial costs) Bold and red = lowest scoring cigarette per country

Underlined = highest score per item across countries

Likert-Scale Ratings: 5-point; Not at all effective (1) to Very effective (5)

Current = Current Packaging Warnings

Cig 1 = Minutes of Life Lost

Cig 2 = Effect of Smoking on Others 2nd overall

Cig 3 = Risk of Mortality from Smoking

Cig 4 = Risk of Addiction from Smoking

Cig 5 = Financial Cost of Smoking 1st overall

Cig 6 = Social Issues with Smoking Last in 3 of 4 countries

Cig 7 = Dealing with Cravings

Cig 8 = Planning to Quit

OpinHW = Opinion (from Strongly Disagree to Strongly Agree) of health warnings and messages being included on all cigarette sticks

Supplementary Appendix 2. Exact numbers of Indigenous participants from each participating country.

**<u>Australia:</u>**	<u>Eleven (11) total</u>
	Seven (7) Aboriginal participants
	One (1) Torres Strait Islander participant
	One (1) Aboriginal/Torres Strait Islander participant
**<u>Canada:</u>**	<u>Eleven (11) total</u>
	Three (3) African American participants
	Eight (8) Native Canadian participants
**<u>UK:</u>**	<u>Nine (9) total</u>
	Seven (7) Black British participants
	Two (2) Afro-Caribbean participants
**<u>USA:</u>**	<u>Eight (8) total</u>
	Eight (8) African American participants

© 2019 Drovandi A.
